# Scaling EPS yield and bio-carrier application of *Bacillus paralicheniformis* UB08 for rapid lead remediation

**DOI:** 10.7717/peerj.21647

**Published:** 2026-08-17

**Authors:** Kaninnut Sangkhum, Thanawan Panich-pat, Pongrawee Nimnoi, Siriporn Wannachat, Kathawut Sopalun, Ratchanee Mingma, Jintanart Wongchawalit

**Affiliations:** 1 Department of Science and Bioinnovation, Faculty of Liberal Arts and Sciences, Kasetsart University, Nakhon Pathom, Thailand; 2 Department of Animal Science, Faculty of Agriculture at Kamphaeng Saen, Kasetsart University, Nakhon Pathom, Thailand

**Keywords:** *Bacillus paralicheniformis*, Extracellular polymeric substances (EPS), Lead adsorption, Cell immobilization, Alginate beads, Bioremediation, Heavy metal

## Abstract

**Background:**

Heavy metal pollution from industrial operations poses severe environmental risks due to its toxicity and bioaccumulation. Conventional chemical and physical remediation methods are costly and generate secondary toxic sludge. Therefore, this study aims to investigate the potential of extracellular polymeric substance (EPS) producing bacteria as a sustainable, eco-friendly alternative for bioremediation.

**Methods:**

EPS producing bacteria were isolated from chemical factory effluent sludge and screened on modified Winogradsky’s medium. The isolates were identified by 16S rRNA gene sequencing and phylogenetic analysis. Heavy metal tolerance to lead (Pb), cadmium (Cd), and mercury (Hg) was evaluated in tryptic soy broth (TSB) containing up to 9,000 ppm of each metal. Culture conditions, including carbon/nitrogen sources, pH, and temperature, were optimized to maximize biomass and EPS yield. Functional groups of EPS were characterized by Fourier transform infrared (FTIR) spectroscopy. To assess bioremediation efficiency, the bacterial isolate was immobilized and encapsulated on ceramic rings, water hyacinth stems, and alginate beads. The surface morphology of the bio-carriers was visualized using field-emission scanning electron microscopy (FESEM). Pb removal efficiency (15 ppm) was quantified using flame atomic absorption spectrophotometry (FAAS).

**Results:**

Among the isolated strains, the most potent strain, *Bacillus paralicheniformis* UB08 (TISTR 10842), displayed an extraordinary nominal Pb tolerance, with a minimum bactericidal concentration (MBC) exceeding 9,000 ppm. In contrast, the strain remained highly sensitive to Cd and Hg with MBC of seven ppm and four ppm, respectively. Optimization in modified TSB (MTSB), containing 0.6% sucrose and 4% yeast extract at pH 6.0 and 55 °C, enhanced EPS production 5.5-fold to 0.4524 ± 0.0176 g/100 ml compared with 0.0821 ± 0.0076 g/100 ml in standard TSB. EPS was characterized as a glycoprotein containing 35.92% glucose and 60.08% fructose equivalents (quantified relative to their respective standards) and 1.35% protein, with a polysaccharide backbone enriched with hydroxyl and amine functional groups. FESEM imaging verified successful biofilm formation and cell immobilization on all matrices. In bioremediation tests, free cells achieved 33.9% of Pb removal. Notably, alginate bead-encapsulated UB08 achieved 100% Pb removal within 2 days, significantly better than immobilization on water hyacinth (100% at 5 days) and ceramic rings (71.51% at 7 days).

**Conclusions:**

* B. paralicheniformis* UB08 demonstrates exceptional thermotolerance and highly efficient Pb removal from solution. Alginate bead encapsulation enables rapid and complete Pb removal, offering a promising solution for heavy metal treatment based on physical adsorption and potential biological synergies.

## Introduction

Water is a fundamental resource essential to sustaining ecological balance, human health, and economic development, and is a critical component of agricultural, industrial, and domestic activities worldwide. Rapid global population growth, urbanization, and industrial expansion have significantly intensified global water demand, leading to increased environmental pollution and deterioration of aquatic ecosystems. Industrial effluents frequently contain hazardous substances that exceed natural safety limits, posing serious risks to ecosystems and public health (Sawyer, McCarty & Parkin, 2003). Among these pollutants, heavy metals such as lead (Pb), cadmium (Cd), and mercury (Hg) are of particular concern due to their extensive industrial use in plastics, paints, batteries, and pharmaceuticals, as well as in agricultural fertilizers and pesticides. Unlike organic pollutants, heavy metals are non-biodegradable and exhibit a strong propensity for bioaccumulation and biomagnification throughout the food web (Salhi et al., 2025).

According to the World Health Organization (2022), chronic exposure to Pb can result in neurological impairment, anemia, and carcinogenic effects, whereas Cd exposure is associated with renal dysfunction and Itai-Itai disease. While Hg is recognized as a potent neurotoxin responsible for Minamata disease, significant heavy metal contamination has been documented in Thailand, including Cd in Tak Province, Pb in Kanchanaburi, and Hg in Rayong (Paijitprapapon, 2003; Suwatvitayakorn et al., 2019; Tremlova, 2017).

Conventional treatment technologies, such as chemical precipitation, electrolysis, and membrane filtration, have been extensively applied to remove heavy metals from wastewater. These physicochemical approaches, however, are frequently constrained by high operational costs, incomplete metal removal, and the generation of secondary toxic sludge requiring further handling and disposal. In contrast, biological treatment methods have emerged as sustainable and eco-friendly alternatives, offering benefits such as reduced costs, high selectivity, and minimal secondary pollution (Loosdrecht et al., 2016).

Among the diverse microorganisms used in bioremediation, the bacterial genus *Bacillus* species has shown exceptional potential owing to its metabolic versatility, tolerance of elevated metal concentrations, and capacity to employ multiple detoxification mechanisms, including biosorption, bioaccumulation, and bioprecipitation (Wróbel et al., 2023). Recent research has demonstrated the considerable effectiveness of *Bacillus* species in sequestering heavy metals, especially lead (Pb). Research by Ren et al. (2015) revealed that *Bacillus* sp. PZ1 exhibits a remarkable Pb adsorption capacity, achieving 93.01% efficiency. Additionally, Shameer (2016) indicated that the EPS of *B. cereus* NSPA8 exhibited similarly high Pb adsorption rates, achieving 90% efficiency. These findings emphasize the significant potential of the genus *Bacillus* for industrial bioremediation applications.

A key factor enhancing microbial resistance to environmental stressors and facilitating heavy metal uptake is EPS production. EPS are complex, high molecular weight biopolymers composed primarily of polysaccharides, proteins, lipids, and extracellular DNA. EPS are secreted by microorganisms to form a protective matrix around cells (Santos et al., 2023). This matrix contributes to structural integrity, promotes cell aggregation and biofilm formation, and provides a protective barrier against toxic compounds. Functionally, EPS contain an array of reactive functional groups, including hydroxyl, carboxyl, phosphate, and amine moieties, which can interact with metal ions through complexation, ion exchange, and electrostatic attraction, thereby supporting metal sequestration and detoxification (Banerjee et al., 2021). EPS-producing bacteria have attracted growing interest as promising agents for the bioremediation of heavy metal contaminated wastewater.

Despite these advantages, the application of EPS-producing bacteria in continuous or large-scale systems is often limited by challenges related to operational stability, biomass retention, and reusability. Free-cell systems can suffer from biomass washout, reduced resistance to hydraulic and toxic shocks, and declining performance over time (Velkova et al., 2018). Cell immobilization on suitable support materials has been proposed as an effective strategy to improve biomass retention and mechanical stability, thereby enhancing remediation efficiency.

In scientific research, porous materials characterized by high structural stability, such as silica gel, activated charcoal, and polyurethane foam, are widely utilized for microbial immobilization due to their expansive specific surface areas and strong physical and chemical bonding capabilities. However, contemporary research has increasingly focused on shifting toward sustainable, cost-effective alternatives to reduce environmental impact (Çabuk et al., 2006; Chengzhou et al., 2009; Utrilla et al., 2001).

Therefore, this study aims to isolate and characterize potent EPS-producing strains exhibiting significant resistance to Pb toxicity, optimize their immobilization on three support materials, and evaluate their efficacy in Pb removal from synthetic wastewater. This study integrates microbial EPS production with immobilization strategies to establish a biologically based model for the sustainable remediation of heavy metal contaminated environments, providing insights into the practical application of such systems in engineered wastewater treatment processes.

## Materials & Methods

### Isolation and screening of EPS-producing bacteria

Sludge samples were collected from the wastewater treatment facility of the chemical manufacturing plant in Rayong Province, Thailand. To isolate indigenous bacteria, serial dilutions of the sludge were performed in sterile distilled water, followed by spread plating on nutrient agar (NA) (HiMedia, India). Plates were incubated at 37 °C for 24 h, and individual colonies were purified *via* the cross-streaking method. The morphological characteristics of the isolated colonies were observed, and Gram staining was performed to identify the Gram reaction.

EPS producing bacterial isolates were screened on Modified Winogradsky’s nitrogen free mineral medium agar (MWA) (containing 0.5% mineral mixed solution medium (5% KH_2_PO_4_, 0.1% MgSO_4_ 7H_2_O, 2.5% NaCl, 0.1% FeSO_4_ 7H_2_O, 0.1% Na_2_MoO_4_ 2H_2_O and 0.1% MnSO_4_ 4H_2_O), 1% Sucrose, 0.005% yeast extract, 0.01% CaCO_3_ and 2% agar, pH 6.2) (Hara et al., 2009) and incubated at 37 °C for 24 to 48 h. EPS-producing isolates were selected based on mucoid phenotypic characteristics, including colorless, transparent, sticky, mucilaginous, elastic, and highly viscous or dome-like colony morphology (Seeoob et al., 2015). This screening strategy, based on the mucoid mode of colonies, follows an established preliminary method for identifying potent EPS producers as described by Fusconi & Godinho (2002). Confirmed EPS-producing strains were collected and preserved in 20% glycerol and stored at −80 °C for further characterization.

### Identification of EPS-producing bacteria by 16S rRNA gene sequences

Genomic DNA was extracted using the phenol-chloroform method. Bacterial cells from an 18 h culture in nutrient broth (NB) were harvested by centrifugation. Mechanical lysis was performed using a Bead Ruptor 24 homogenizer (Omni International) at a speed of four m/s for 30 s (1 cycle) with glass beads. Subsequently, the lysate was subjected to heat shock at 100 °C for 10 min. Phenol chloroform isoamyl alcohol (25:24:1, v/v) (Shamash, Kapoor & Maurice, 2025) was added to the cell lysate. The DNA layer was collected after centrifugation, and precipitated with cold absolute ethanol, then washed with 70% ethanol. The genomic DNA was resuspended in TE buffer and incubated with RNase at 37 °C for 30 min.

The 16S rRNA gene was amplified by polymerase chain reaction using 16S rRNA universal primers 27F (5′-AGAGTTTGATCCTGGCTCAG-3′, forward primer) and 1525R (5′-AAAGGAGGTGATCCAGCC-3′, reverse primer) (Wongchawalit, Noitanom & Panichpat, 2020) with MyTaq™ Red Mix 2X (Meridian Bioscience, USA). PCR products with 1,500 bp DNA targets were verified by agarose gel electrophoresis. Nucleotide sequences of PCR products were determined using barcode-tagged sequencing and analyzed using the NCBI BLAST nucleotide tool (https://blast.ncbi.nlm.nih.gov/Blast.cgi) to determine taxonomic identity. Sequence alignment and phylogenetic tree construction were performed using MEGA11 software version 11.0.13 (Elrashedy et al., 2025).

### Minimum bactericidal concentration determination

The *B. paralicheniformis UB08* suspensions in Tryptic Soy Broth (TSB) (HiMedia, India) were standardized to a 0.5 McFarland turbidity standard (approximately 1.5 × 10^8^ cells/ml). A 10% (v/v) inoculum was then introduced into TSB supplemented with various concentration ranges of heavy metals, including Pb(NO_3_)_2_ (1–9,000 ppm), Cd(NO_3_)_2_ (1–6,000 ppm), and HgSO_4_ (1–6,000 ppm). After incubation at 37 °C with shaking at 150 rpm for 24 h, a loopful of each culture was subcultured onto Tryptic Soy Agar (TSA) (HiMedia, India) plates, followed by further incubation at 37 °C for 24 h. The minimum bactericidal concentration (MBC) was defined as the lowest concentration of heavy metal that completely inhibited visible bacterial growth on the agar plates.

### Optimization of culture conditions for bacterial growth and EPS production

A systematic optimization of culture conditions was performed to maximize both bacterial biomass and EPS yield. Growth curves (OD_600_) and yields of biomass and EPS were initially compared in NB and TSB for 96 h.

For biomass and EPS quantification, cultures were centrifuged at 10,000 rpm, 4 °C for 20 min. The cell pellet was collected for biomass measurement, and the EPS was precipitated from the supernatant using pre-cooled isopropanol at a 2:1 (v/v) ratio. The mixture was centrifuged at 10,000 rpm and 4 °C for 20 min to recover EPS precipitates. Both bacterial cell pellets and EPS precipitates were subsequently lyophilized (Christ, Osterode am Harz, Germany) and weighed to determine the dry weight of biomass and EPS. Based on the results, the medium provided the highest biomass yield, and EPS production was used as the basal medium to further optimize the parameters for bacterial growth and EPS production.

#### Effect of carbon source types and concentrations

Modified TSB (MTSB) medium (2% casein peptone, 0.5% NaCl, 0.25% KH_2_PO_4_) was prepared in 100 ml and supplemented with 0.6% (w/v) of one of the following carbon sources, including glucose, sucrose, maltose, fructose, or lactose. The pH of each medium was adjusted to 7.0. The bacterial inoculum (10%, v/v) was inoculated into the medium and incubated at 37 °C with shaking at 150 rpm for 24 h. Biomass and EPS were harvested, lyophilized, and quantified as dry weight using the previous method.

The carbon source that yielded the highest EPS production was selected for further concentration optimization. MTSB medium (100 ml) was then supplemented with a variation of the best carbon source concentration at 0.2%, 0.4%, 0.6%, 0.8%, or 1.0% (w/v) and adjusted to pH 7.0. Bacterial culture (10% v/v) was inoculated into the medium and incubated under identical conditions. Biomass and EPS production yield were recorded as dry weight.

#### Effect of nitrogen source types and concentrations

MTSB medium 100 ml containing the optimal carbon source and concentration from previous experiments was supplemented with 2% (w/v) of one of the following nitrogen sources, including peptone, urea, casein peptone, yeast extract, ammonium nitrate (NH_4_NO_3_), ammonium sulfate ((NH_4_)_2_SO_4_), or ammonium chloride (NH_4_Cl). Each medium was adjusted to pH 7.0 prior to sterilization, then inoculated with 10% (v/v) bacterial inoculum and incubated at 37 °C with shaking at 150 rpm for 24 h. Biomass and EPS production yields were determined.

The nitrogen source yielding the highest EPS production was selected for concentration optimization. MTSB medium (100 ml) was prepared with the optimal carbon source and this nitrogen source at 0.5%, 1%, 2%, 3%, or 4% (w/v), adjusted to pH 7.0. The 10% (v/v) of the bacterial inoculum was inoculated and cultivated under identical conditions. Biomass and EPS were harvested, lyophilized, and quantified in milligrams dry weight.

#### Effect of pH

MTSB medium (100 ml) containing the optimal carbon and nitrogen sources and concentrations from previous experiments was adjusted to pH 5.0, 6.0, 7.0, 8.0, or 9.0. Each medium was inoculated with 10% (v/v) bacterial culture and incubated at 37 °C with shaking at 150 rpm for 24 h. Biomass and EPS were quantified as dried weight.

#### Effect of temperatures

The optimal pH was then used to evaluate the effect of temperatures. MTSB medium (100 ml) was prepared with the optimal carbon source, nitrogen source, and pH, inoculated with 10% (v/v) bacterial culture, and incubated at 30, 37, 45, or 55 °C with shaking at 150 rpm for 24 h. Biomass and EPS were harvested, lyophilized, and quantified as dried weight.

### Quantification of growth rate and EPS production under optimized conditions in MTSB

The growth rate, biomass, and EPS yield of the selected isolate were evaluated under optimized conditions. The bacterial inoculum was standardized in MTSB (McFarland 0.5), and 10% v/v was inoculated into 100 ml of MTSB with the previously determined optimal carbon/nitrogen sources and pH. To determine the growth rate, the culture was shaken and incubated at 37 °C and 150 rpm. The optical density at 600 nm was measured hourly for 96 h to generate the growth curve. For EPS and biomass quantification, the culture was collected every 24 h for 4 days. At each time point, cell pellets were obtained by centrifugation, and EPS was extracted from the supernatant by isopropanol precipitation. Briefly, the EPS was precipitated by adding isopropanol pre-chilled at −20 °C at a 2:1 (v/v) ratio, and the precipitation process was allowed to proceed overnight at 25 °C. The precipitated EPS was then collected by centrifugation at 12,000 rpm and 4 °C for 20 min, followed by dialysis through a cellulose membrane with a flat width size of 30/32 against distilled water for 48 h. Finally, the dialyzed sample was lyophilized at −55 °C under a vacuum pressure of 0.125 mbar for 48 h. Both bacterial cell pellets and EPS precipitates were lyophilized and weighed to determine the dry weight of biomass and EPS.

### EPS extraction and preliminary purification

The cell-free supernatant was mixed with chilled isopropanol at a 2:1 (v/v) ratio and allowed to precipitate at room temperature. The mixture was centrifuged at 12,000 rpm (4 °C) to collect the crude EPS. Protein removal was performed using Sevag’s reagent (chloroform:1-butanol, 4:1 v/v) according to Fan et al. (2015). The crude EPS was redissolved in DI water, mixed with Sevag’s reagent at a 1:2 (v/v) ratio, and stirred overnight at 4 °C. The mixture was transferred to a separatory funnel and left at room temperature for at least 2 h to allow phase separation. After discarding the lower organic phase, the upper aqueous phase containing the purified EPS was collected. This phase was then dialyzed using a cellulose membrane (MWCO 12,000–16,000 Da) against 2,000 ml of DI water for 48 h at room temperature with continuous stirring. Finally, the dialyzed EPS was dried in a lyophilizer at −55 °C under 0.128 mbar for 16 h (or until completely dry) to obtain the purified EPS powder.

### Characterization of EPS

#### Total sugar and total protein composition

Total carbohydrate content was determined by the phenol sulfuric acid method modified from Chaplin (1994) and Dubois et al. (1956). The two hundred microliters of EPS sample (50 µg/ml) or standards (glucose and fructose, 0–60 µg/ml) were mixed with 200 µl of 5% (w/v) phenol and 1 ml of concentrated H_2_SO_4_. After incubation, absorbance was measured at 490 nm. Sugar content was quantified using both glucose and fructose standard curves.

Protein content was quantified using the Bradford assay (Bradford, 1976; Caprette, 2015). Bovine serum albumin (BSA) standards were prepared (at concentrations 0–1.0 mg/ml). Each standard concentration (20 µl) or EPS samples (20 µl) was mixed with one ml Bradford reagent (Coomassie Brilliant Blue G-250). Samples were vortexed and incubated at room temperature for 5 min. Absorbance was measured at 595 nm, and protein concentrations were determined against a BSA standard curve.

#### Chemical composition and functional groups of EPS by Fourier transform infrared spectroscopy

Dried EPS samples were analyzed by Fourier transform infrared (FTIR) spectroscopy (Spectrum Two; PerkinElmer) over 4,000–600 cm^−1^ at two cm^−1^ resolution with 32 scans. Spectra were acquired and analyzed using PerkinElmer Spectrum software to identify characteristic absorption bands of functional groups.

### Bacterial immobilization and encapsulation on support materials for Pb-remediation assays

#### Carrier preparation

Three support materials, including ceramic rings, water hyacinth (*Eichhornia crassipes*) stems, and alginate beads, were used for bacterial immobilization and encapsulation. Alginate beads were prepared using a 2% w/v sodium alginate solution, which was dropped from a 50 ml syringe into 2% w/v CaCl_2_ solution. The beads were continuously stirred in the solution using a magnetic stirrer for 30 min to ensure crosslinking, resulting in a uniform bead size of approximately 0.4 cm. Additionally, the water hyacinth stems were pretreated by drying at 55 °C and cut into segments with a diameter of approximately two cm, while the ceramic rings used had a standardized diameter of approximately 2.2 cm.

#### Free cells

The heavy metal-tolerant, high EPS-producing bacterial isolate was cultivated in MTSB medium under previously optimized conditions. MTSB medium (270 ml) was inoculated with 10% (v/v) seed culture and incubated at 37 °C, 150 rpm for 24 h (OD600 approximate 1.0). Cells were harvested by centrifugation at 8,000 rpm, 4 °C for 15 min. The pellet was subsequently resuspended for Pb remediation assays.

#### Ceramic ring and water hyacinth stem immobilization

The bacterial inoculum 10% (v/v) was inoculated into 270 ml of MTSB medium containing sterile ceramic rings (approximately 18 g) and sterilized water hyacinth stems (approximately 12 g). The culture was then incubated at 37 °C, 150 rpm for 24 h. Bacteria immobilized on ceramic rings and water hyacinth stems were recovered by filtration through sterile cheesecloth for further Pb remediation assay.

#### Alginate bead encapsulation

MTSB medium (270 ml) was inoculated with 10% (v/v) seed culture and incubated at 37 °C, 150 rpm for 24 h to reach an OD600 of approximately 1.0. Bacterial cells were harvested by centrifugation at 8,000 rpm, 4 °C for 15 min. To achieve uniform cell loading, the entire harvested cell pellet obtained from the 270 ml culture was thoroughly resuspended and homogenized in 250 ml of sterile 2% (w/v) sodium alginate solution. The homogeneous cell-alginate suspension was then added dropwise into 250 ml of a sterile 2% (w/v) CaCl_2_ solution using a 50 ml syringe to form the encapsulated alginate beads. All immobilized and encapsulated preparations were immediately utilized for Pb remediation assays.

### Scanning electron microscopy characterization

The surface morphology and structural distribution of bacterial cells on the support matrices were visualized using Field Emission Scanning Electron Microscopy (FESEM) (JEOL). Following the immobilization process, the ceramic rings, water hyacinth stalks, and alginate beads were desiccated in a hot air oven at 70 °C until a constant weight was achieved. For sample preparation, the ceramic and water hyacinth stalks were sectioned into thin transverse slices (<5 mm), and the alginate beads were bisected to expose their internal architecture. All samples were mounted onto aluminum stubs and sputter-coated with platinum to enhance conductivity. Structural characterization was performed at varying magnifications to evaluate bacterial attachment, biofilm formation, and the integrity of the immobilization matrices.

### Pb removal efficiency of immobilized bacteria by flame atomic absorption spectrophotometry (FAAS) analysis

#### Synthetic wastewater containing Pb and experimental design

Synthetic wastewater (250 ml) containing 15 ppm Pb was prepared by 0.0024% (w/v) lead nitrate (Analytical Reagent grade, Qrec, Auckland, New Zealand) in water and autoclaved. The Pb removal assay was evaluated across eight treatments as follows: (C) sterile control, (B) free cells of isolate UB08, (CC) control ceramic rings, (CB) ceramic rings immobilized with isolate UB08, (HC) control water hyacinth stalks, (HB) water hyacinth stalks immobilized with isolate UB08, (AC) control alginate beads, and (AB) alginate beads encapsulated with isolate UB08. Each material was added to each flask and incubated at 37 °C and 150 rpm.

#### Sampling and sample preparation

Daily 25 ml aliquots were collected from all treatments over 7 days and acidified with 5 ml concentrated HNO_3_. Pb solution at concentrations of 0.5–15 ppm was prepared as a standard. Digestion of all samples and standards was performed using a Digestor 2520 (FOSS) at 95 °C. The process was continued for 4–6 h or until a final volume of 5–10 ml remained.

Following digestion, each sample was filtered (Whatman No. 42), and the final filtrate volume was adjusted to 25 ml with distilled water prior to AAS analysis (Varian).

#### FAAS analysis

Pb concentrations were quantified by FAAS at 283.3 nm with background correction. The Pb hollow cathode lamp was warmed for 30 min, and the instrument was autozeroed with Type 1 distilled water. A calibration curve was constructed using standards at 0.5–15 ppm (R^2^ approx. 1.0), providing a Limit of Detection (LOD) of 0.05 ppm to ensure the accuracy and reliability of the quantified data. Sample absorbance values were converted to concentrations by the instrument software. The percentage of residual Pb was subsequently calculated using the following formula.

(1) \begin{eqnarray*}\%~\text{Pb remaining}= \frac{\text{Concentration of Pb remaining}}{\text{Initial Pb concentration}} \times 100\end{eqnarray*}

(2) \begin{eqnarray*}\%~\text{Pb removal}=100-\%\text{Pb remaining}.\end{eqnarray*}

### Data analysis

Data were obtained from three independent experiments and are presented as mean ± SD. Significant differences among groups were analyzed by one-way analysis of variance (ANOVA), and multiple comparisons were performed using Tukey’s Honestly Significant Difference (HSD) test.

## Results

### Isolation, morphological characterization, and identification of EPS producing bacteria

A total of 26 bacterial isolates, designated with isolation codes from UB02 through UB33, were recovered from the chemical industrial wastewater sludge. Gram staining and microscopic examination revealed a predominance of gram-positive bacteria (20 isolates) over gram-negative bacteria (six isolates). Morphological screening on MWA medium identified 13 EPS-producing isolates were identified by their characteristic hyaline, mucoid, and convex colony morphology. Within the 13 EPS-producing isolates, nine were gram-positive, and four were gram-negative.

**Table 1:** Identification of bacterial isolates based on 16S rRNA gene sequence analysis. Percent identified was determined by comparing 16S rRNA gene sequences against the NCBI database using the BLAST algorithm. Accession numbers correspond to deposition codes in public databases.

**Code** ** number**	**Accession** ** number**	**Organism name**	**Percent** ** identified**
UB02	SRR32886099	*Aeromonas caviae*	99.93
UB03	SRR32886098	*Stenotrophomonas acidaminiphila*	99.93
UB04	SRR32886088	*Bacillus cereus*	100.00
UB05	SRR32886087	*Mammaliicoccus sciuri*	100.00
UB06	SRR32886086	*Exiguobacterium arabatum*	100.00
UB07	SRR32886085	*Priestia aryabhattai*	100.00
UB08	SRR32886084	*Bacillus paralicheniformis*	100.00
UB09	PZ596610	*Bacillus cereus*	100.00
UB10	SRR32886082	*Pseudomonas khazarica*	99.04
UB11	PZ596606	*Bacillus paralicheniformis*	100.00
UB12	PZ596611	*Bacillus cereus*	100.00
UB13	PZ596607	*Bacillus paralicheniformis*	100.00
UB14	PZ596612	*Bacillus cereus*	100.00
UB15	SRR32886081	*Bacillus rugosus*	100.00
UB16	PZ596613	*Bacillus cereus*	100.00
UB20	PZ596608	*Bacillus paralicheniformis*	100.00
UB23	PZ596609	*Bacillus paralicheniformis*	100.00
UB24	SRR32886097	*Staphylococcus warneri*	99.91
UB25	SRR32886096	*Bacillus tropicus*	99.93
UB26	SRR32886095	*Achromobacter xylosoxidans*	99.24
UB27	SRR32886094	*Bacillus paramycoides*	100.00
UB29	SRR32886093	*Ectopseudomonas hydrolytica*	99.87
UB30	SRR32886092	*Pseudomonas hydrolytica*	99.87
UB31	SRR32886091	*Bacillus paramobilis*	100.00
UB32	SRR32886090	*Bacillus tropicus*	99.93
UB33	SRR32886089	*Bacillus paramycoides*	100.00

**Figure 1:**
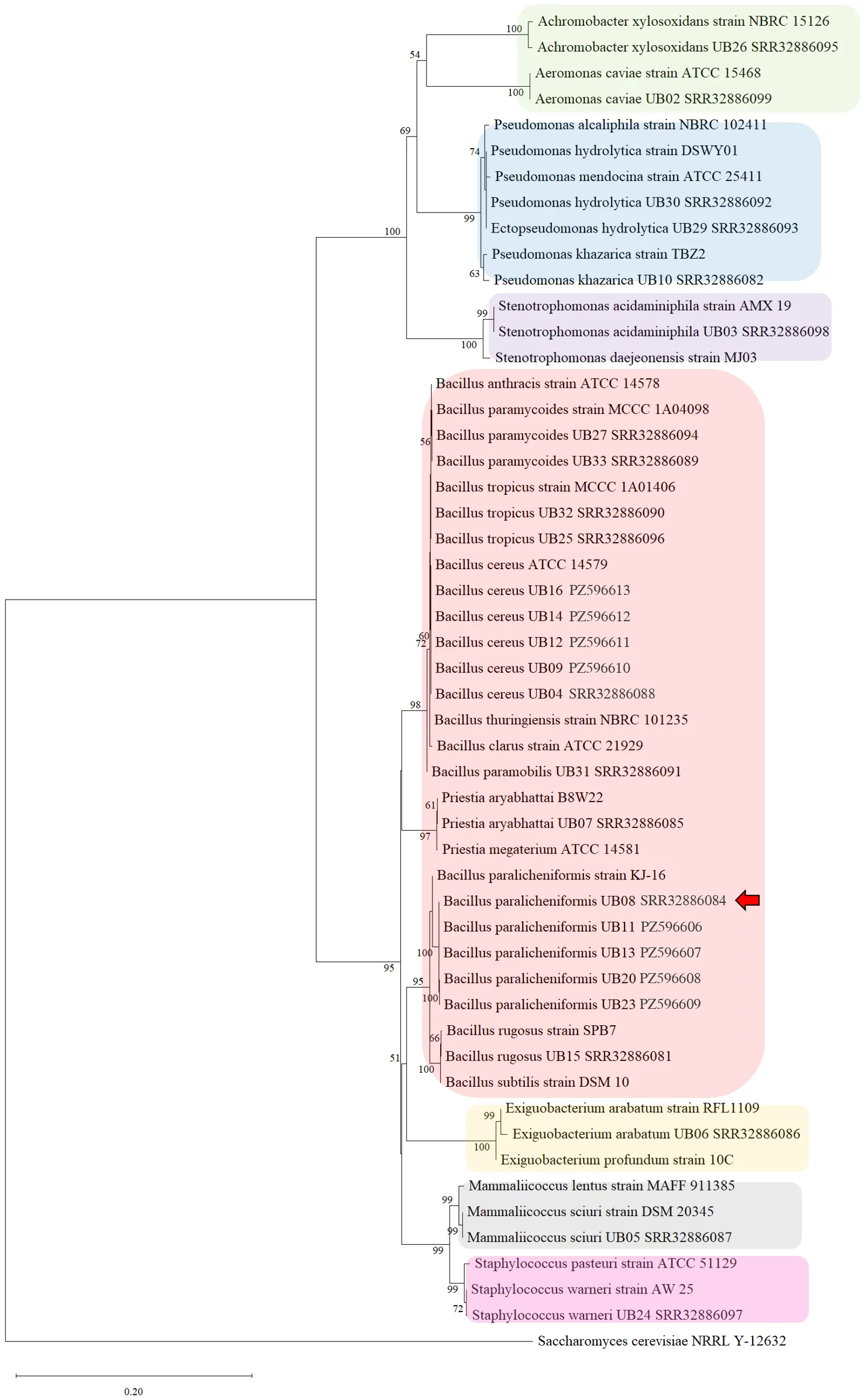
Molecular phylogenetic analysis of bacterial strains isolated from activated sludge based on 16S rRNA gene sequences. The analysis involved various bacterial genera, including *Bacillus*, *Pseudomonas*, *Stenotrophomonas*, *Achromobacter*, *Aeromonas*, *Exiguobacterium*, *Mammaliicoccus*, and *Staphylococcus*, which were isolated from activated sludge. *Saccharomyces cerevisiae* NRRL Y-12632 was used as the outgroup. The red arrow highlights the specific placement of *Bacillus paralicheniformis* UB08 (SRR32886084) within the *Bacillus* cluster.

**Figure 2:**
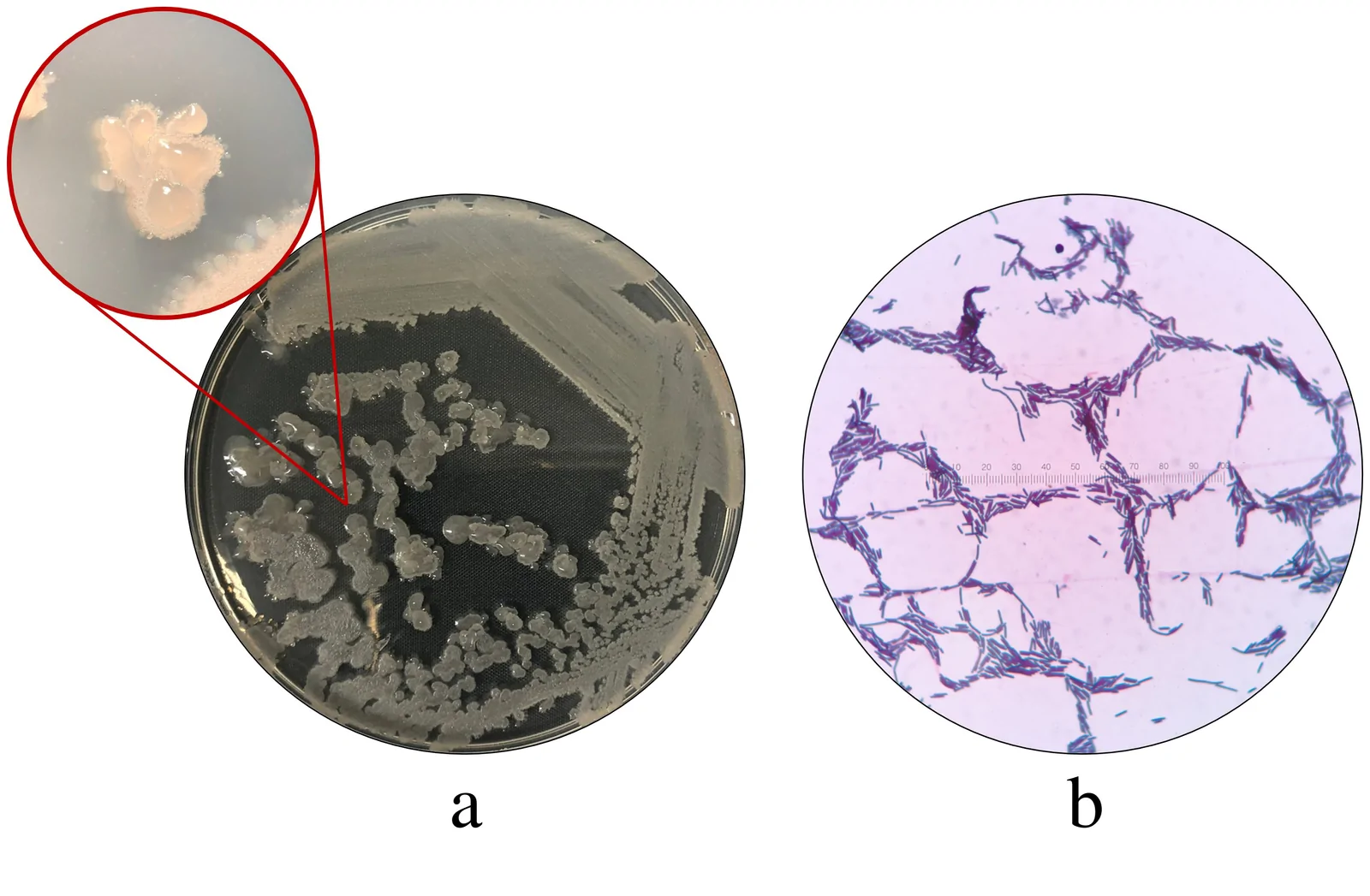
Morphological and microscopic characteristics of *B paralicheniformis* UB08. (A) Colony morphology of *B. paralicheniformis* UB08 on Nutrient Agar (NA) after 24 h of incubation at 37 °C. The colonies appear mucoid, opaque, and cream-colored with irregular margins. (B) Gram staining and microscopic observation of the isolate (1,000x magnification). The strain UB08 is identified as a Gram-positive, rod-shaped bacterium.

The 16S rRNA gene sequences from the 26 isolates were compared with the GenBank database using the BLASTn tool for taxonomic identification. All 26 isolates were successfully identified to the species level, with a sequence identity expectation of 99%. This analysis revealed a diverse microbial community comprising 19 distinct bacterial species (Table 1). To further elucidate the genetic relationships among these isolates, a phylogenetic tree was constructed (Fig. 1). The resulting phylogeny demonstrated significant microbial diversity, with isolates distributed among multiple major clades in conjunction with their corresponding reference strains. The tree was supported by high bootstrap values approaching 100%, indicating strong statistical confidence in the branching patterns. Correlating with this phylogenetic grouping, the quantitative distribution detailed in Table 1 showed that the genus *Bacillus* accounted for the largest percentage (61.53% of the 26 isolates). The other predominant genera identified across the clades included *Achromobacter, Aeromonas, Pseudomonas, Stenotrophomonas, Priestia, Exiguobacterium, Mammaliicoccus,* and *Staphylococcus*. Among these, isolate UB08 was identified as *B. paralichenifor mis* (100% identity), a Gram-positive bacterium that exhibited the highest EPS production capacity. This was evidenced by its colonies displaying a distinct, viscous, glossy, and highly mucoid appearance (Fig. 2). The raw 16S rRNA gene sequencing reads were deposited in the NCBI Sequence Read Archive (SRA) under accession number SRR32886084. The strain *B. paralicheniformis* UB08 was deposited in the Thailand Institute of Scientific and Technological Research (TISTR) under the accession number TISTR 10842.

### Heavy metal tolerance capacities of *B. paralicheniformis* UB08

**Figure 3:**
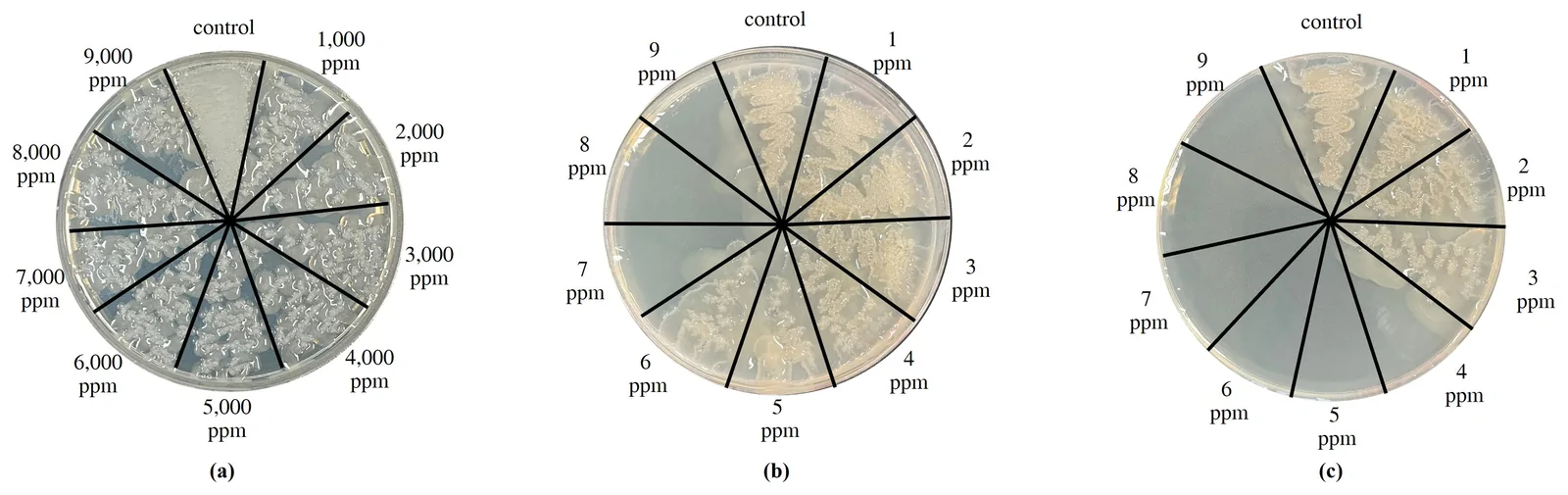
Minimum bactericidal concentration (MBC) determination of *Bacillus paralicheniformis* UB08. *B. paralicheniformis* UB08 after 24 h of exposure to different heavy metals, subcultured on TSA plates. The assays show cell viability profiles against: (A) lead (Pb(NO_3_)_2_) ranging from 1,000 to 9,000 ppm, (B) cadmium (Cd(NO_3_)_2_) ranging from one to nine ppm, and (C) mercury (HgSO_4_) ranging from one to nine ppm. Control sectors represent bacterial growth in metal-free media. All assays were performed in triplicate (*n* = 3).

**Figure 4:**
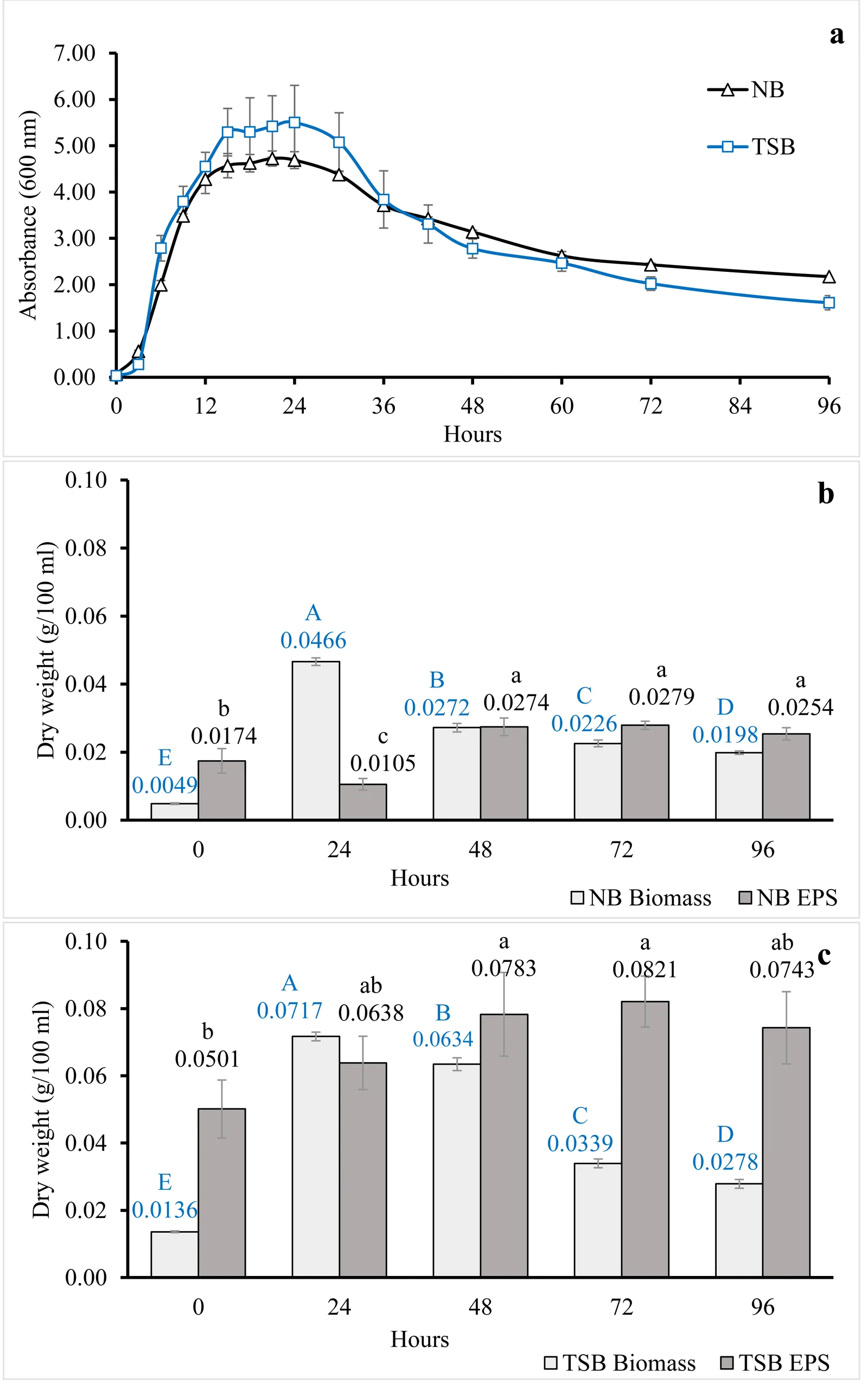
Growth and EPS production of *Bacillus paralicheniformis* UB08 in NB and TSB media. (A) Growth curves of strain UB08 in NB and TSB media during incubation at 37 °C and 150 rpm for 96 h, monitored by optical density at 600 nm. (B) Biomass and EPS production in NB medium at 0–96 h. (C) Biomass and EPS production in TSB medium at 0–96 h. All assays were performed in triplicate (*n* = 3).

The tolerance of *B. paralicheniformis* UB08 was evaluated by measuring growth after culturing in TSB containing different concentrations of Pb, Cd, and Hg and subculturing onto TSA plates. Table 2 and Fig. 3 reveal that *B. paralicheniformis* UB08 formed colonies at 0 ppm (control) for all metals. *B. paralicheniformis* UB08 exhibited remarkable resistance to Pb ions exceeding 9,000 ppm (Fig. 3A), whereas Cd and Hg exhibited total growth inhibition at 7 ppm (Fig. 3B) and 4 ppm (Fig. 3C), respectively, with no colony formation observed at these concentrations (Figs. 3B and 3C, respectively). Based on these initial quantitative screening results summarized in Table 2 and Fig. 3, *B. paralicheniformis* UB08 exhibited distinct minimum bactericidal concentrations (MBC) for each heavy metal, with the highest tolerance observed against Pb. Consequently, further optimization and bioremediation efficiency experiments were conducted exclusively using Pb.

**Table 2:** Minimum bactericidal concentrations (MBC) of various heavy metals against *B. paralicheniformis* UB08. All assays were performed in triplicate (*n* = 3) following a 24 h incubation at 37 °C in TSB containing heavy metal, with subsequent subculture on Tryptic Soy Agar (TSA) plates.

**Heavy metal**	**MBC (ppm)**
Pb(NO_3_)_2_	>9,000
Cd(NO_3_)_2_	7
HgSO_4_	4

### Optimization of growth and EPS production in *B. paralicheniformis* UB08

Optimization of growth and EPS production in *B. paralicheniformis* UB08 was achieved through stepwise evaluation of medium composition and culture conditions. Figure 4A showed a classical four-phase growth curve. Comparative growth in NB and TSB revealed that TSB supported a significantly higher maximum cell density (OD_600_ max 5.503 at 24 h) than NB (OD_600_ max 4.723 at 21 h). TSB also produced the greatest biomass (0.0717 ± 0.0013 g/100 ml), with significantly higher EPS production observed on day 3 at 0.0821 ± 0.0076 g/100 ml (Figs. 4B and 4C). Consequently, TSB was selected as the basal medium (MTSB) for further optimization.

**Figure 5:**
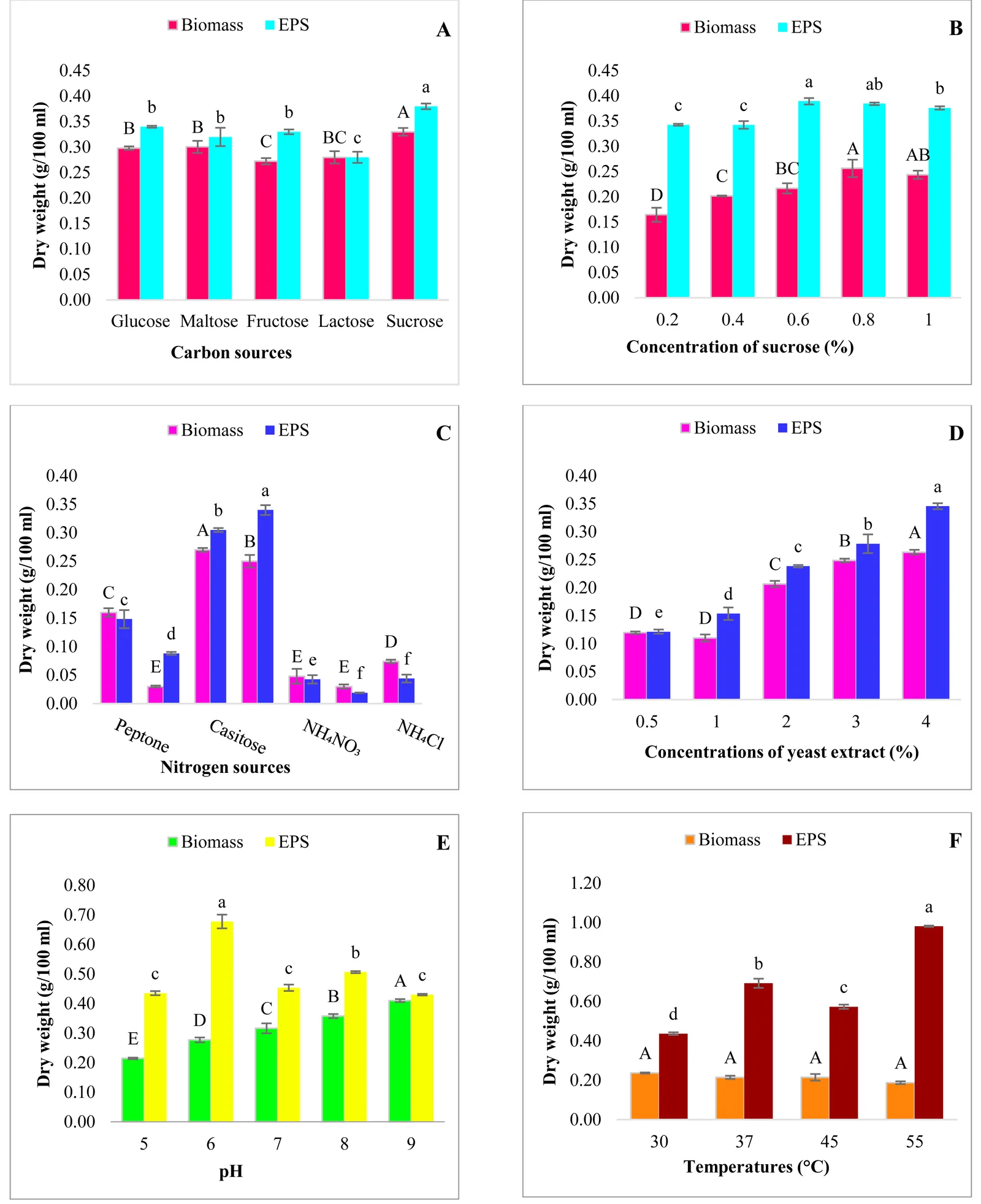
Optimization of culture conditions for biomass and EPS production by *Bacillus paralicheniformis* UB08. (A) Effect of different carbon sources on biomass and EPS yield. (B) Optimization of sucrose concentrations ranging from 0.2% to 1.0% (w/v). (C) Effect of different nitrogen sources on biomass and EPS yield. (D) Optimization of yeast extract concentrations ranging from 0.5% to 4.0% (w/v). (E) Effect of pH (5.0−9.0) on biomass and EPS production. (F) Effect of incubation temperatures (30–55 °C) on biomass and EPS production. All assays were performed in triplicate (*n* = 3).

The effects of nutritional and physicochemical parameters, including carbon and nitrogen sources, pH, and temperature, on growth and EPS production in MTSB were demonstrated in Fig. 5. Among the carbon sources tested, Fig. 5A demonstrated that sucrose was the best carbon source for biomass and EPS production and at a concentration of 0.6% (w/v) was found to be the most significant effective, yielding 0.3894 g/100 ml of EPS (Fig. 5B). Nitrogen source screening identified yeast extract as the optimal nitrogen source (Fig. 5C), increasing yeast extract concentration at 4% (w/v) enhanced the maximum EPS of 0.3457 g/100 ml (Fig. 5D). Environmental factors also played a critical role, biomass was highest at alkaline pH 8–9, maximum EPS production of 0.6774 g/100 ml was achieved at pH 6 (Fig. 5E). Notably, Temperature profiling indicated that elevated temperatures strongly promoted EPS synthesis, with the highest yield of 0.9811 g/100 ml at 55 °C (Fig. 5F).

**Figure 6:**
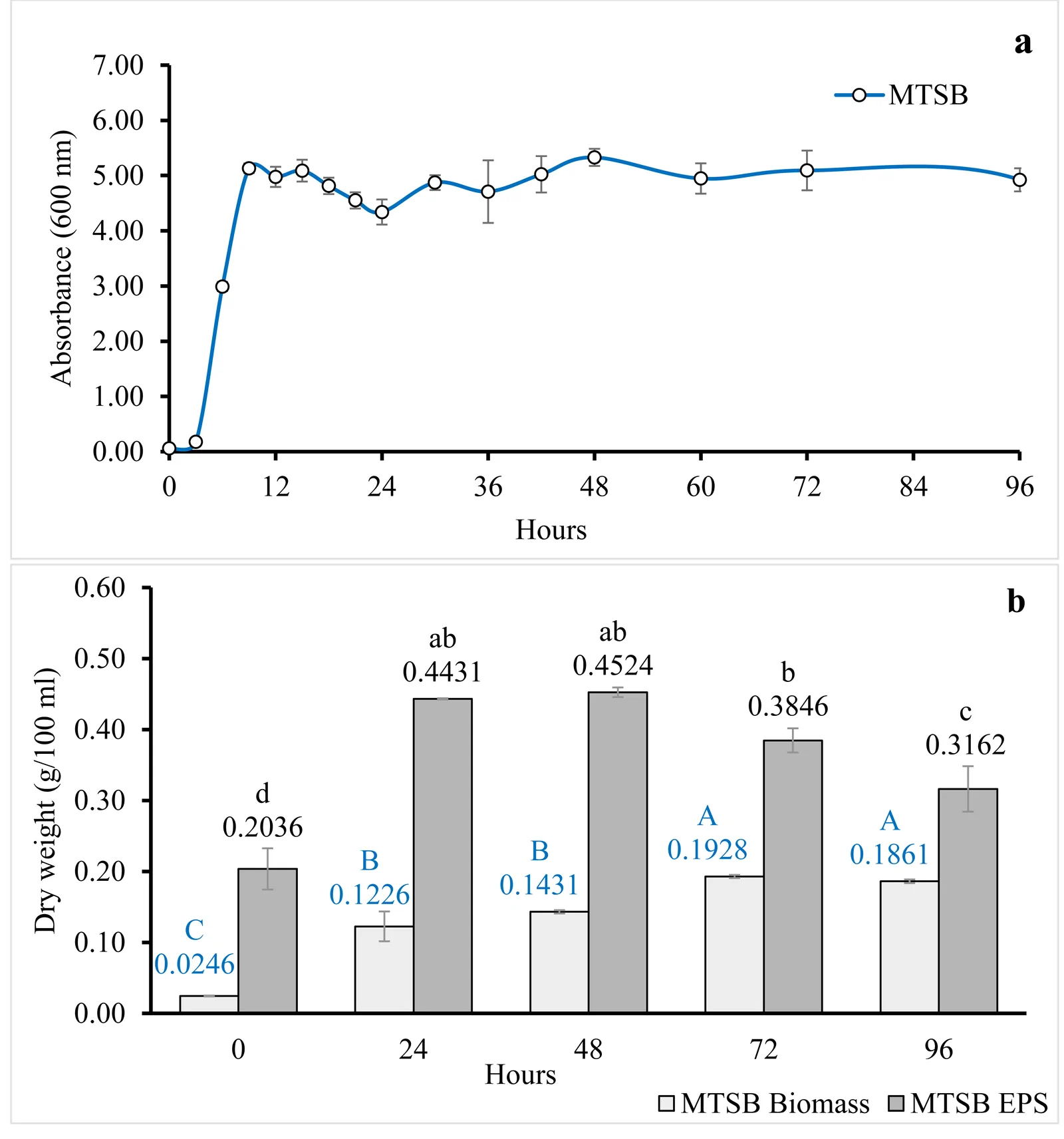
Growth and EPS production in Modified TSB (MTSB). (A) Growth curve of *B. paralicheniformis* UB08 in MTSB medium during incubation at 37 °C and 150 rpm for 120 h, monitored by optical density at 600 nm. (B) Biomass and EPS production in MTSB medium over 96 h. All assays were performed in triplicate (*n* = 3).

Under fully optimized conditions, TSB was modified to MTSB, containing 0.6% sucrose, 4% yeast extract, and adjusted to pH 6. For the final optimized cultivation, the incubation temperature was set at 37 °C. The growth and EPS production of *B. paralicheniformis* UB08 were again investigated, and the results are shown in Fig. 6. The bacterial strain exhibited significantly accelerated exponential growth peaking at 9 h (OD_600_ at 5.13 ± 0.10) (Fig. 6A). Biomass reached its maximum of 0.1928 ± 0.0021 g/100 ml within Days 3, whereas EPS production peaked earlier on Day 2, yielding 0.4524 ± 0.0176 g/100 ml (Fig. 6B).

### Chemical composition and functional group analysis of EPS

The chemical composition of purified EPS from *B. paralicheniformis* UB08 was analyzed. Using the phenol-sulfuric acid method, the total sugar content was determined to be 17.96 µg/ml (35.92% w/w) and 30.04 µg/ml (60.08% w/w) when quantified against glucose and fructose standard curves, respectively. Furthermore, the Bradford assay showed a low protein content of 0.2023 mg/ml, representing only 1.35% of the EPS dry weight.

**Figure 7:**
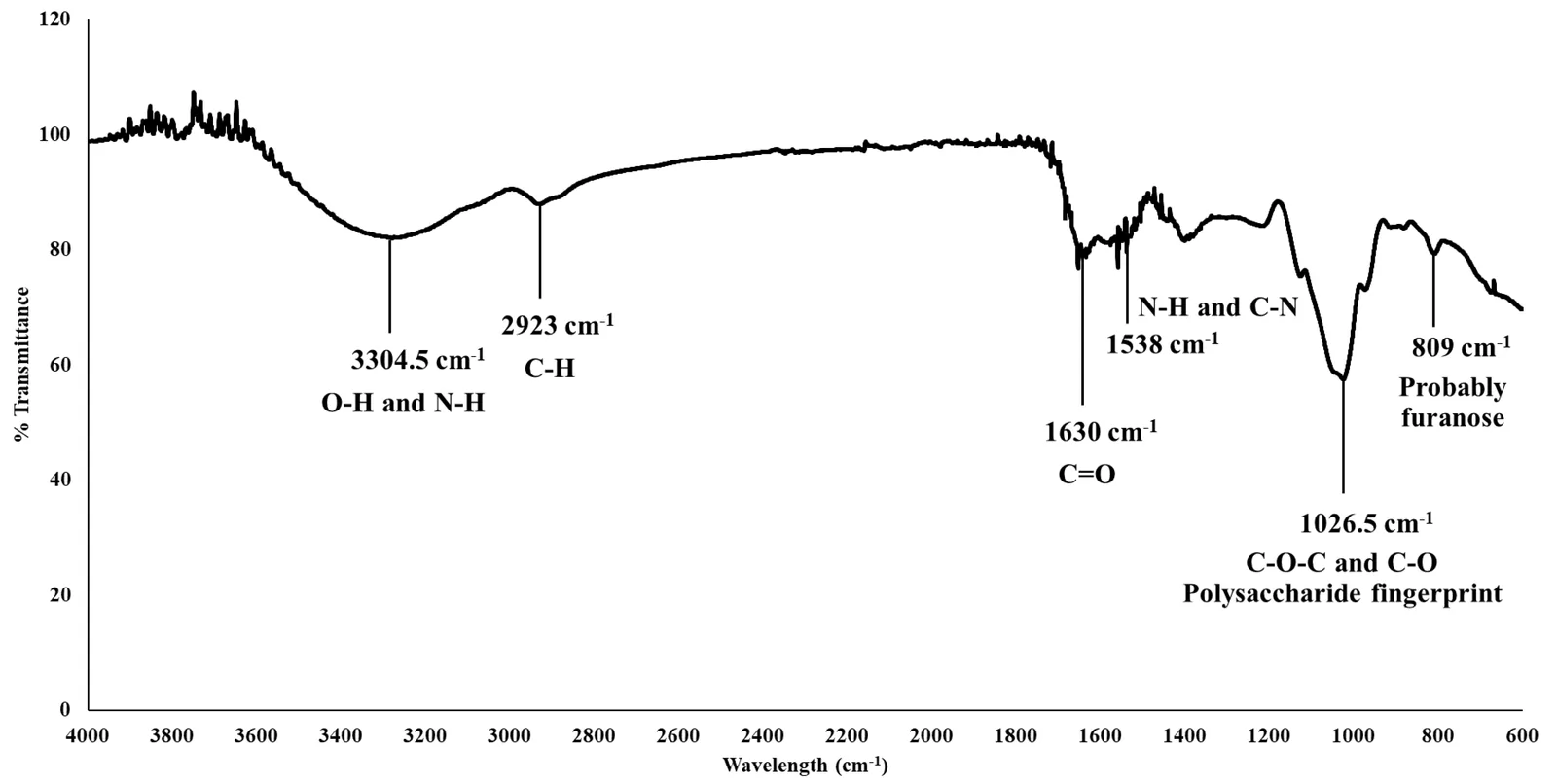
FTIR spectrum of EPS from *B. paralicheniformis* UB08. The spectrum was recorded from 4,000 to 600 cm^−1^ at a resolution of 2 cm^−1^ with 32 scans.

The structural configuration and functional groups of the EPS were further elucidated through FTIR spectroscopy. A broad, intense absorption band at 3,304.5 cm^−1^ corresponded to O-H and N-H stretching vibrations, indicating an abundance of hydroxyl and amine groups. Aliphatic C-H stretching was observed at 2,923 cm^−1^, confirming the presence of methyl and methylene groups. The proteinaceous component was further evidenced by the detection of Amide I (1,630 cm^−1^, C=O stretching) and Amide II (1,538 cm^−1^, N-H bending and C-N stretching) bands. A prominent fingerprint peak at 1,026.5 cm^−1^, attributed to C-O-C and C-O stretching in glycosidic linkages, confirmed the dominance of a polysaccharide backbone. Furthermore, the emergence of a distinct band at 809 cm^−1^ is characteristic of C-H vibrations in furanose rings (Fig. 7).

### FESEM analysis of bacterial immobilization and encapsulation on support materials

**Figure 8:**
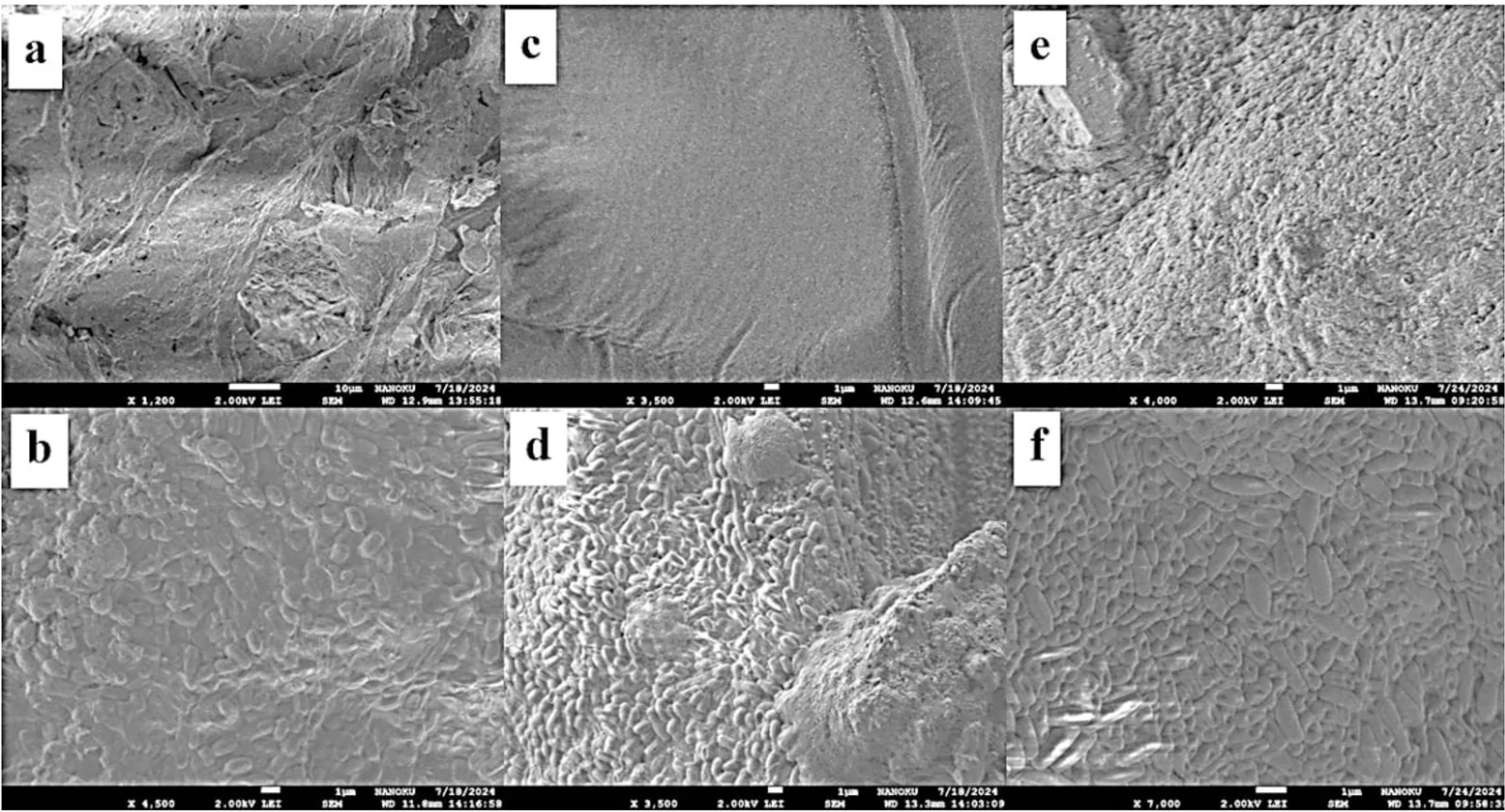
FESEM of *B. paralicheniformis* UB08 immobilization on various support matrices. (A), (C) and (E) show the morphology of the ceramic ring, water hyacinth stem, and alginate bead, respectively. (B), (D) and (F) show morphology after immobilization.

FESEM imaging confirmed the successful colonization and biofilm formation of *B. paralicheniformis* UB08 across all three support matrices, including ceramic rings, water hyacinth stems, and alginate beads (Fig. 8).

Prior to immobilization, the surfaces of the ceramic rings and water hyacinth stalks appeared relatively smooth and lacked microbial life (Figs. 8A, 8C, respectively). After immobilization, both matrices exhibited a dense, coarse coating of rod-shaped bacterial cells embedded within an EPS matrix, indicative of biofilm development (Figs. 8B, 8D).

For encapsulation, the alginate beads exhibited the most significant morphological change. The initially irregular, porous abiotic surface (Fig. 8E) was entirely transformed into a uniform, densely packed layer of encapsulated EPS producing bacterial cells (Fig. 8F).

### Pb removal efficiency of immobilized bacteria

The experiment demonstrates effective Pb bioremediation by the *B. paralicheniformis* UB08 bacterium across various immobilization matrices, as measured by flame atomic absorption spectrometry (FAAS). The Pb removal capacities were evaluated through eight treatments in synthetic wastewater containing 15 ppm of Pb.

**Figure 9:**
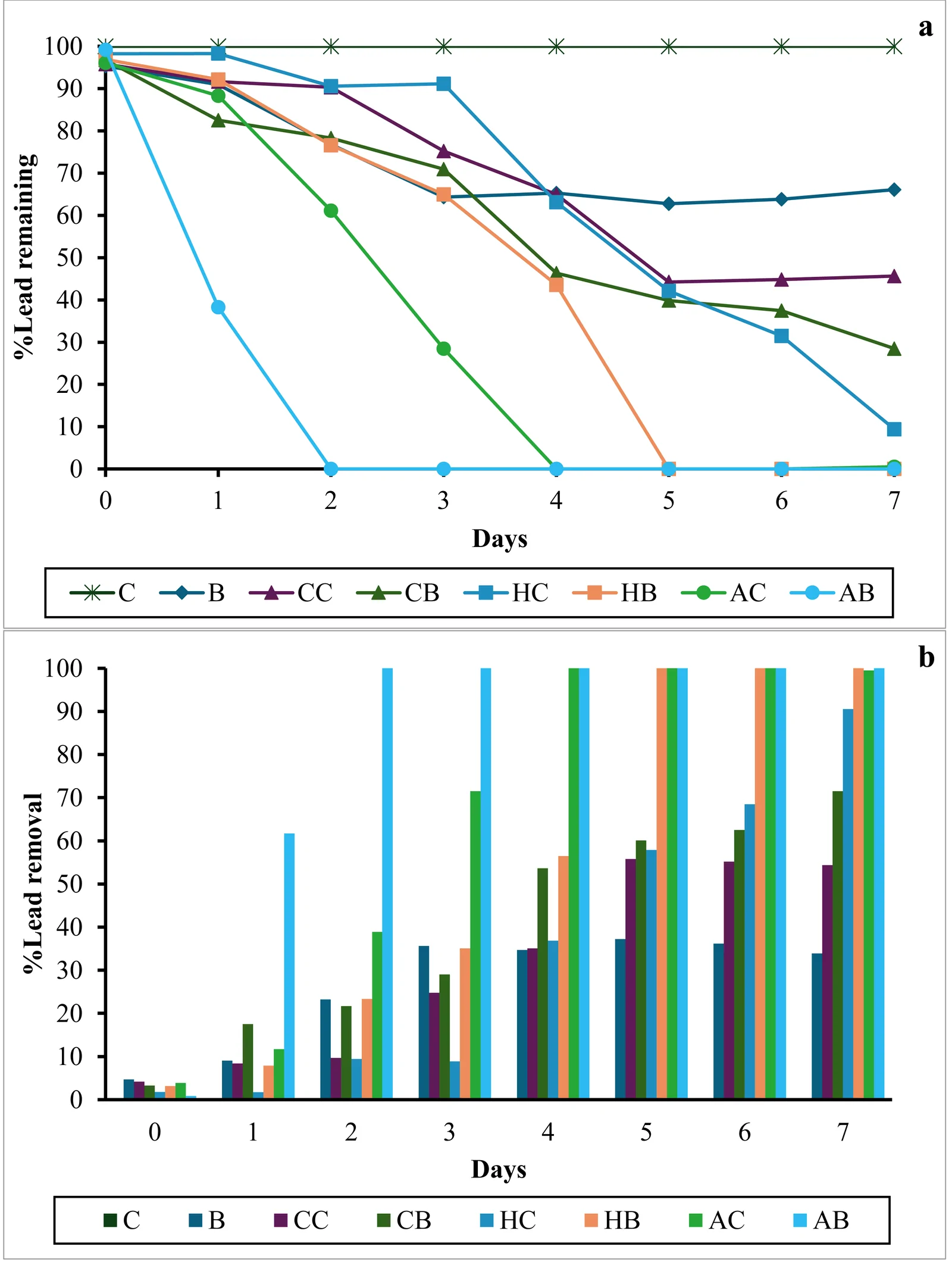
Lead removal efficiency by immobilized *B. paralicheniformis* UB08. The experiment was conducted with an initial Pb concentration of 15 ppm over 7 days. (A) Percentage of lead remaining in the solution. (B) Percentage of lead removal efficiency. Treatments include C, Control; B, Free cells of UB08; CC, Control ceramic rings; CB, Ceramic rings immobilized with UB08; HC, Control water hyacinth stalks; HB, Water hyacinth stalks immobilized with UB08; AC, Control alginate beads; AB, Alginate beads encapsulated with UB08. All assays were performed in triplicate (*n* = 3).

In terms of lead remaining, as shown in Table 3 and Fig. 9A, Pb concentrations in treatment C remained constant at 15 ppm (100% remaining) over the 7-day period. In comparison, free cells isolate UB08 (treatment B) showed limited efficiency on their own, leaving Pb at 9.91 ppm (66.10% remaining) at the end of the 7-day period. Treatment AC showed 0 ppm (0% remaining) on day 4. In contrast, treatments CC and HC remained at 6.84 ppm (45.63% remaining) and 1.41 ppm (9.42% remaining), respectively. Immobilized treatments, including HB and AB, reached 0.00 ppm (0% remaining). In particular, AB reduced Pb to 0.00 ppm within 2 days.

**Table 3:** Lead remaining concentration in the Pb solution (15 ppm) during the 7-day removal.

Treatments	Lead remaining (mean ± standard deviation) (ppm)							
	Day 0	Day 1	Day 2	Day 3	Day 4	Day 5	Day 6	Day 7
C	15.00 ± 0.00^a^	15.00 ± 0.00^a^	15.00 ± 0.00^a^	15.00 ± 0.00^a^	15.00 ± 0.00^a^	15.00 ± 0.00^a^	15.00 ± 0.00^a^	15.00 ± 0.00^a^
B	14.30 ± 0.25^a^	13.64 ± 0.20^bcd^	11.51 ± 0.09^c^	9.65 ± 0.15^e^	9.79 ± 0.17^b^	9.41 ± 0.34^b^	9.57 ± 0.31^b^	9.91 ± 0.48^b^
CC	14.37 ± 0.00^a^	13.74 ± 0.64^abc^	13.54 ± 0.33^b^	11.28 ± 0.15^c^	9.74 ± 0.33^b^	6.63 ± 0.11^c^	6.72 ± 0.31^c^	6.84 ± 0.36^c^
CB	14.51 ± 0.11^a^	12.37 ± 0.60^d^	11.74 ± 0.32^c^	10.64 ± 0.33^d^	6.95 ± 0.44^c^	5.98 ± 0.26^c^	5.62 ± 0.33^d^	4.27 ± 0.22^d^
HC	14.73 ± 0.40^a^	14.74 ± 0.00^ab^	13.58 ± 0.55^b^	13.67 ± 0.26^b^	9.47 ± 0.30^b^	6.31 ± 0.33^c^	4.72 ± 0.16^e^	1.41 ± 0.20^e^
HB	14.53 ± 0.27^a^	13.82 ± 0.14^abc^	11.49 ± 0.44^c^	9.74 ± 0.09^e^	6.53 ± 0.23^c^	0.00 ± 0.00^d^	0.00 ± 0.00^f^	0.00 ± 0.00^f^
AC	14.41 ± 0.60^a^	13.24 ± 0.44^cd^	9.16 ± 0.11^d^	4.27 ± 0.16^f^	0.00 ± 0.00^d^	0.00 ± 0.00^d^	0.00 ± 0.00^f^	0.07 ± 0.00^f^
AB	14.87 ± 0.34^a^	5.74 ± 0.26^e^	0.00 ± 0.00^e^	0.00 ± 0.00^g^	0.00 ± 0.00^d^	0.00 ± 0.00^d^	0.00 ± 0.00^f^	0.00 ± 0.00^f^

**Notes.**

C Control B Free cells of UB08 CC Control ceramic rings CB Ceramic rings immobilized with UB08 HC Control water hyacinth stalks HB Water hyacinth stalks immobilized with UB08 AC Control alginate beads AB Alginate beads encapsulated with UB08

All assays were performed in triplicate (*n* = 3). Statistical differences between treatment means were analyzed using One-way Analysis of Variance (ANOVA), followed by Tukey’s Honestly Significant Difference (HSD) test at a 95% confidence level (*p* < 0.05), where distinct lowercase letters (a–g) indicate significant differences between experimental groups.

Hence, based on the % removal as shown in Fig. 9B, the control remained at 0% removal throughout the 7-day experiment, indicating no intrinsic sorption by the medium alone. Free UB08 cells (B) showed only a gradual increase in removal, reaching roughly 30–35% by Day 7. The treatments with support materials alone, as well as treatments CC, HC, and AC, exhibited varying degrees of passive adsorption. Treatment AC achieved complete 100% Pb removal by day 4, while treatment HC and CC removed concentrations of Pb at 90.58% and 54.37%, respectively, by day 7. Interestingly, cell immobilization significantly synergized remediation rates. *B. paralicheniformis* UB08 encapsulated in alginate beads (AB) achieved 100% Pb^2+^ removal within only 2 days, while cells immobilized, HB reached complete removal by day 5. Ceramic treatments showed an intermediate pattern, with removal rising more slowly than alginate and hyacinth, but still reaching about 71.51.% removal by Day 7.

## Discussion

In this study, we focused on screening and isolating EPS-producing bacteria and evaluating their ability to immobilize or encapsulate on ceramic rings, dried water stalks, and alginate beads for the bioremediation of heavy metals. The microbial diversity in the industrial sludge revealed a community dominated by genera such as *Achromobacter*, *Pseudomonas*, and *Bacillus*. The use of such mucoid phenotypic characteristics as a primary selection criterion is a well recognized approach for identifying potent EPS producing bacteria from environmental samples, as it directly reflects the accumulation of exopolymers around the bacterial colonies (Fusconi & Godinho, 2002). The prevalence of *Bacillus* species, specifically the identification of isolate UB08 as *B. paralicheniformis*, underscores their suitability for bioremediation. Bacteria in this group are Gram-positive bacilli capable of producing endospores and exhibit remarkable resistance to extreme environmental stressors, such as high temperatures, dryness, and radiation. This capacity allows *B. paralicheniformis* to thrive in heavy-metal contaminated areas more effectively than non-spore-forming bacteria (Wróbel et al., 2023). It is commonly found in extreme environments such as deserts, mines, and marine habitats, with strain TB197 from the Mexican desert producing pest-inhibiting metabolites during drought stress (Quicaño et al., 2023), and strain BL-1 achieved phenol degradation in high-salinity environments at up to 10% NaCl (Zhang et al., 2025).

Previous findings support the classification of the genus *Bacillus* as a highly adaptable taxon capable of maintaining viability despite significant physiological stressors (Krajanglikit et al., 2018). In addition to overall adaptability, *Bacillus* species exhibit strong resistance to heavy metals, providing continued microbial activity in contaminated environments (Liu et al., 2024).

According to Shah (2017), these heterotrophic bacteria are pivotal in biodegradation and nutrient cycling within wastewater systems. Consequently, the utilization of bacteria to remediate heavy metal contaminated water sources is a compelling approach. Previous investigations have reported significant Pb tolerance levels, notably *Streptomyces toxytricini* FM1 and *Bacillus cereus* ID1, which demonstrated resistance limits of 5,200 ppm and 5,180 ppm, respectively (Idrees et al., 2023; Bakran, Aly & Zabermawi, 2019). Additionally, industrially relevant strains such as *Pseudomonas chlororaphis* Hel-KE-14 and *Raoultella planticola* FACU 3 showed significantly lower tolerance levels at 2,800 ppm and 2,700 ppm, respectively (Elarabi et al., 2023; Khaled, Helal & Ali, 2016).

However, our findings indicated that *B. paralicheniformis* UB08 exhibited a high nominal lead tolerance, with an MBC value exceeding 9,000 ppm. This strong resistance profile highlights its potential suitability for heavy metal bioremediation applications. This outstanding capacity identifies UB08 as a highly promising candidate for heavy metal bioremediation. This resistance is likely facilitated by EPS production, which forms a biopolymer matrix. Those matrices serve as a protective shield, sequestering metal ions through complexation with hydroxyl, amine, and carboxyl functional groups before their potential intracellular toxicity (Qiao et al., 2019). Although intracellular distribution and specific heavy metal resistance or transport genes (such as the *pbrA* gene or metallothionein complexes) were not characterized in this current study (Jarosławiecka & Piotrowska-Seget, 2014), the prominent extracellular entrapment by hydroxyl and amino functional groups on the EPS matrix, as characterized by our FTIR analysis, is hypothesized to serve as a supportive frontline barrier. This physicochemical mechanism potentially restricts excessive toxic influx into the intracellular space, ensuring cell viability and high lead tolerance (Alotaibi, Khan & Shamim, 2021). Further genetic profiling and cellular tracking will be pursued in future molecular-level investigations. Focusing the subsequent remediation and optimization assays solely on Pb, while excluding Cd and Hg, is consistent with the strain’s selective tolerance profile. Bacillus species typically exhibit different survival thresholds depending on the cellular detoxification mechanisms activated by individual divalent ions (Alotaibi, Khan & Shamim, 2021). In this study, the high MBC observed for Pb, together with the marked sensitivity to Cd and Hg (Table 2), suggests that the metabolic and remediative capacity of *B. paralicheniformis* UB08 is strongly skewed toward Pb. Specifically, the strain demonstrated extreme sensitivity to Cd and Hg, with MBC established at seven ppm and four ppm, respectively. This distinct toxicity profile is consistent with the higher intrinsic cytotoxicity of Cd and Hg ions, which often disrupt cell membrane integrity and inhibit essential enzymatic pathways at much lower concentrations than lead (Alotaibi, Khan & Shamim, 2021). By defining these precise low-concentration thresholds, the operational boundaries of strain UB08 can be clearly delineated for targeted environmental applications. Therefore, testing different lead concentrations with this strain is a rational strategy for developing practical environmental applications. In addition, heavy metal stress induced elevated extracellular EPS production, which created a technical limitation for standard growth monitoring. The resulting non-cellular colloidal turbidity interfered with spectrophotometric readings and prevented reliable tracking by OD_600_. For this reason, the quantitative MBC values confirmed by viable colony recovery (Table 2 and Fig. 3) were used as a more dependable measure of the strain’s tolerance under different heavy metal loads. Nonetheless, these literature comparisons should be perceived as a general indicator of bioremediation potential rather than a direct parallel, as variations in initial metal concentrations, culture media, and experimental designs inevitably vary across different studies.

The optimization of culture conditions revealed that TSB significantly surpassed NB, possibly due to TSB having a more varied nutrient composition, including peptides, amino acids, and vitamins that provide necessary metabolic precursors for growth and secondary metabolite biosynthesis. Our findings indicated that sucrose was the best carbon source for biomass and EPS production. This observation is consistent with previous studies reporting that *Bacillus paralicheniformis* prefers sucrose as a carbon source for EPS synthesis, specifically in the form of levan, a homopolymer of fructose. This preference aligns with the levansucrase pathway, in which the enzyme levansucrase, typically encoded by the *sacB* gene, catalyzes the conversion of sucrose into levan, and *sacB* expression is markedly upregulated when sucrose is present in the culture medium. The use of sucrose as the primary carbon source also functions as an inducer for *sacB* gene expression *via* positive regulation. As sucrose concentrations increase, the cell perceives this through the phosphotransferase system (PTS), signaling an upregulation of extracellular levansucrase synthesis. Consequently, *B. paralicheniformis* UB08 exhibits superior utilization of sucrose as a primary carbon source compared to other sugars (Lekakarn et al., 2025). This observation corroborates prior studies across *Bacillus* species. For instance, the research by Bashandy et al. (2024) demonstrated that the highest EPS yield (0.47 g/100 ml) from *Bacillus subtilis* was obtained using a medium supplemented with 4% sucrose.

Optimization of carbon sources by Miri et al. (2021) for *Bacillus zhangzhouensis* ZB 16 revealed that sucrose significantly outperformed other sugars, including glucose, fructose, and maltose. Under these optimized conditions, the strain achieved a peak EPS production of 0.795 g/100 ml.

Furthermore, research by Dobara et al. (2014) confirmed that sucrose as a carbon source enables *B. subtilis* to produce significantly higher EPS yield than other carbon substrates.

Yeast extract serves as a highly bioavailable source of both nitrogen and carbon for bacterial uptake. Furthermore, it is rich in essential amino acids and vitamins that significantly promote bacterial growth (Banerjee et al., 2026). Also, polysaccharide production is favored by a high carbon/nitrogen ratio, as with other EPS-producing microorganisms (Miri et al., 2021).

Our findings align with research by Rao et al. (2013), which demonstrated that *Bacillus amyloliquefaciens* BPRGS produced a maximum EPS yield of 2.65 g/100 ml using 5% sucrose and 1% yeast extract. This reinforces the suitability of these substrates for enhancing exopolysaccharide production in our current study. Not only the *Bacillus* strain, but also research on other EPS-producing bacteria, yielded results consistent with ours finding. Dailin et al. (2022) identified 10.6% sucrose and 3.0% yeast extract as the optimal concentrations for EPS production in *Lactobacillus reuteri*, reaching a maximum yield of 0.34 g/100 ml. This further validates the use of these components in our MTSB medium to promote superior metabolic activity and EPS secretion.

Additionally, the pH of the culture medium serves as a critical factor in modulating enzymatic activity. According to the study by Xavier & Ramana (2017), *Bacillus licheniformis* ANT 179 is capable of synthesizing EPS primarily composed of levan. This biosynthetic process is mediated by the enzyme levansucrase, which exhibits optimal synthesis at pH 6.0 and 60 °C. These findings are consistent with my current research, as *B. paralicheniformis* UB08 demonstrated EPS production at pH 6.0 and 55 °C. This aligns with the levansucrase hypothesis, where the enzyme responsible for synthesizing levan-type EPS common in *Bacillus* species exhibits maximal activity at slightly acidic pH levels (Phengnoi et al., 2022).

Finally, the optimized condition in MTSB medium enhanced EPS production 5.5-fold to 0.4524 ± 0.0176 g/100 ml, compared with 0.0821 ± 0.0076 g/100 ml produced in standard TSB. This significant increase is attributed to the secondary-metabolite nature of EPS, typically associated with the early stationary phase (Saha & Datta, 2022). The abundance of sucrose and yeast extract in MTSB accelerated the growth rate of *B. paralicheniformis* UB08, leading to a higher biomass yield and reaching a higher stationary phase level than those observed in NB and TSB. Consequently, these optimized growth conditions directly correlated with the increased EPS yields. It should be noted that the One-Factor-at-a-Time (OFAT) approach used in this study does not account for interactive effects among factors. Future work will employ multi-factorial optimization (*e.g.*, Response Surface Methodology) to identify global optima and synergistic interactions during scale-up. Notably, the difference between the maximum yield during screening (0.9811 g/100 ml at 55 °C) and the final cultivation (0.4524 ± 0.0176 g/100 ml at 37 °C) represents an intentional choice. To optimize energy sustainability and practical bioremediation scaling, 37 °C was preferred over 55 °C for the final system despite the lower absolute EPS yield.

Preliminary chemical and structural characterization revealed that the EPS was a glycoprotein containing glucose and fructose equivalents (35.92% and 60.08%, respectively), along with 1.35% protein, confirming its identity as a hydrophilic biopolymer. The remaining 2.65% of the dry weight is attributed to residual moisture or trace organic constituents. FTIR spectra corroborated the presence of O-H, N-H, and C=O groups, which are critical for the chelation of heavy metals. Importantly, this observed functional profile of the UB08 EPS aligns closely with the established FTIR benchmarks for the *Bacillus* genus. The major absorption bands recorded in this study including the O-H/N-H stretching (3,304.5 cm^−1^), aliphatic C-H stretching (2,923.0 cm^−1^), Amide I (1,630.0 cm^−1^), Amide II representing N-H bending and C-N stretching (1,538.0 cm^−1^), and the prominent C-O-C/C-O stretching (1,026.5 cm^−1^), are highly consistent with the characteristic ranges reported for other potent EPS-producing *Bacillus* strains, such as *B. subtilis*, *B. licheniformis*, and *B. cereus* (Banerjee et al., 2018; Marimuthu & Rajendran, 2023; Rehman, Vrouwenvelder & Saikaly, 2021). Furthermore, the structural configuration of the polysaccharide backbone was corroborated by the C-H vibrations of the furanose ring at 809.0 cm^−1^, a unique signature that strongly mirrors the fructan-type exopolymers documented in related literature (Marimuthu & Rajendran, 2023). These findings align with previous studies on *B. paralicheniformis* from various sources. For instance, Nasir et al. (2022) identified levan as the primary EPS produced by a strain isolated from buffalo grass, and similarly, Halmouch et al. (2023) reported that *B. paralicheniformis* ND12 from the Mediterranean Sea also yielded levan. These functional groups not only facilitate water retention but also provide a high density of anionic sites for Pb binding (Biswas et al., 2020).

To enhance lead adsorption efficiency, ceramic rings, dried hyacinth stalks, and alginate beads have emerged as promising options, representing inorganic, lignocellulosic, and biopolymeric material classes, respectively. These materials are environmentally friendly and exhibit diverse structural properties and functional groups that significantly enhance immobilization efficiency while promoting cost-effectiveness and resource valorization (Dutto et al., 2024; Lissy & Madhu, 2011; Wang et al., 2016).

A significant finding of this study was the Pb^2+^ removal efficiency of the immobilized systems. The alginate-encapsulated *B. paralicheniformis* UB08 achieved complete (100%) removal of 15 ppm Pb^2+^ within 48 h, surpassing both free cells and abiotic supports. While complete Pb removal was observed, this enhanced efficiency is tentatively attributed to a synergistic effect between the physical adsorption of the alginate matrix and hypothetical EPS-mediated biosorption or bioprecipitation. However, the precise contribution of each component and the physical location of the sequestered Pb remains to be confirmed.

This performance notably exceeds the results reported by Chatterjee et al. (2015), who achieved 99.5% removal but at a much lower initial lead concentration of one ppm. The ability of encapsulated UB08 to maintain total efficiency at a 15-fold higher metal load underscores the robustness of its levan-rich EPS and the protective environment of the alginate beads. However, it is worth noting that while calcium-alginate provides an excellent biocompatible matrix for rapid remediation, it typically faces structural degradation over extended or multi-cycle operations compared to synthetic alternatives. In this study, we utilized fresh immobilized beads for each test to ensure maximum metabolic activity, but tailoring carrier durability for long-term reusability remains an important goal for future upscaling work. The combined effect of the support matrices and *B. paralicheniformis* UB08 was further clarified by comparing the lead removal efficacy of abiotic control carriers (AC, CC, and HC) with that of the bio-immobilized systems (AB, CB, and HB). The control carriers provided a standard for physical adsorption, but UB08 markedly enhanced removal rates. The alginate control (AC) accomplished 100% removal in 4 days, while the bio-immobilized alginate (AB) achieved the same efficiency in exactly 2 days. This divergence occurs because the abiotic matrix drives direct chemical complexation. In contrast, free cells are directly exposed to lead toxicity. Consequently, their growth and EPS synthesis are suppressed, and the secreted EPS becomes diluted in the medium to be effective. The improved performance is likely enhanced by the biological activity of the EPS layer and biofilm formation (as illustrated in Fig. 8F), which potentially provides additional sequestration sites and promotes bioprecipitation alongside physical adsorption by the carriers. However, without direct post-remediation elemental analysis, this cooperative mechanism remains to be confirmed.

While ceramic rings provided mechanical stability, their inflexible construction nevertheless restricted mass transfer, leading to a 70.57% reduction by Day 7. Conversely, water hyacinth stalks provided a highly porous, cellulose-rich environment that favored biofilm attachment, leading to complete Pb removal by day 5. Given the low cost and availability of water hyacinth as an agricultural waste product, its use as a carrier offers a highly sustainable, circular-economy approach to wastewater treatment (Nurfajriani, 2024). Nevertheless, certain operational limits of this study should be noted. The remediation experiments used a simplified synthetic matrix with a single baseline (15 mg/l Pb^2+^) to optimize parameters. Furthermore, while calcium-alginate is highly biocompatible, its potential for structural degradation, cell leaching, and multi-cycle reusability remains an operational boundary that defines the current batch-scale setup. In complex industrial effluents, higher metal loads (50–100 mg/l) and competing cations (*e.g.*, Ca^2+^, Mg^2+^) can influence biosorption yields by saturating exopolysaccharide-binding sites. Additionally, while FTIR analysis successfully identified the primary functional groups involved, characterizing multi-ion competitive isotherms and incorporating post-remediation analysis SEM-EDX mapping to trace elemental distribution represents valuable prospects for future scale-up investigations.

## Conclusions

This study successfully identified *B. paralicheniformis* UB08 isolated from industrial sludge as an EPS producing strain with a highly potent Pb-resistant bacterium capable of thriving at concentrations up to 9,000 ppm Pb^2+^. Through nutritional and environmental optimization, maximal EPS production was achieved in a sucrose-yeast extract medium at pH 6.0 and 55 °C. Chemical and spectroscopic characterization by FTIR confirmed that EPS is a glycoprotein-rich biopolymer possessing hydroxyl and amine functional groups, which are theoretically capable of heavy metal ligation. Furthermore, the application of cell immobilization techniques significantly enhanced bioremediation efficiency. While abiotic supports and free cells showed limited efficacy, *B. paralicheniformis* UB08 encapsulated in alginate beads achieved total Pb removal within 48 h, followed by water hyacinth-immobilized cells, which reached 100% removal by 5 days. These findings advance biological wastewater treatment by demonstrating that low-cost agricultural waste can serve as an effective carrier for immobilized biosorbents, offering substantial advantages over conventional physicochemical methods in terms of cost, sustainability, and operational efficiency. The performance of this integrated biocatalytic system highlights its strong potential for complete water decontamination. While this study demonstrates total Pb removal from solution, the precise mechanism and exact physical distribution of the sequestered metal on the bio-carriers remain unconfirmed. Therefore, future research must incorporate post-remediation characterization (such as SEM-EDX and ICP-MS analysis of carrier material) to definitively establish the sequestration pathways, alongside evaluating long-term matrix stability, carrier reusability, cell leaching rates, and performance under multi-cycle operations, as well as potential interference from coexisting pollutants, to fully validate its actual field-scale applicability in industrial wastewater treatment.

## Supplemental Information

10.7717/peerj.21647/supp-1 Supplemental Information 1 Raw data

## Additional Information and Declarations

### Data Availability

The following information was supplied regarding data availability: The raw measurements are available in the Supplementary File. The raw sequence data are available at NCBI: PRJNA1242817, SRR32886081–SRR32886099.
